# Trauma-related dissociation and altered states of consciousness: a call for clinical, treatment, and neuroscience research

**DOI:** 10.3402/ejpt.v6.27905

**Published:** 2015-05-19

**Authors:** Ruth A. Lanius

**Affiliations:** Western University, Lawson Health Research Institute, London, ON, Canada

**Keywords:** Dissociation, consciousness, interoceptive awareness, dissociative subtype, emotion, anterior cingulate, insula, complex PTSD

## Abstract

The primary aim of this commentary is to describe trauma-related dissociation and altered states of consciousness in the context of a four-dimensional model that has recently been proposed (Frewen & Lanius, 2015). This model categorizes symptoms of trauma-related psychopathology into (1) those that occur within normal waking consciousness and (2) those that are dissociative and are associated with trauma-related altered states of consciousness (TRASC) along four dimensions: (1) time; (2) thought; (3) body; and (4) emotion. Clinical applications and future research directions relevant to each dimension are discussed. Conceptualizing TRASC across the dimensions of time, thought, body, and emotion has transdiagnostic implications for trauma-related disorders described in both the Diagnostic Statistical Manual and the International Classifications of Diseases. The four-dimensional model provides a framework, guided by existing models of dissociation, for future research examining the phenomenological, neurobiological, and physiological underpinnings of trauma-related dissociation.

Formative work by Janet (1901) identified “dissociation” of traumatic material from consciousness as a central defense against overwhelming experience. Here, dissociation provides a critical psychological escape from emotional and physical distress associated with overwhelming traumatic experience, including childhood maltreatment, war trauma, and torture, from which no actual physical escape is possible (Kluft, 1985; Nijenhuis, Vanderlinden, & Spinhoven, 1998; Putnam, 1996; Spiegel, 1984; Vermetten, Doherty, & Spiegel, 2007; also see Carlson, Yates, & Sroufe, 2009; Liotti, 2009; Schore, 2009). This type of escape can involve *compartmentalization* where “aspects of psychobiological functioning that should be associated, coordinated, and/or linked are not” (Spiegel, 2012; Spiegel et al., 2011, p. E19; also see Van der Hart, Nijenhuis, & Steele, 2006) and *detachment*, including depersonalization, derealisation, and emotional numbing (Allen, 2001; Brown, 2006; Holmes et al., 2005; Spiegel & Cardena, 1991; Steele, Dorahy, Van der Hart, & Nijenhuis, 2009; Van der Kolk & Fisler, 1995). Downstream, however, as an individual attempts to resume normal functioning in the aftermath of trauma, chronic dissociation can have devastating consequences for all aspects of life (Brand et al., 2009; Jepsen, Langeland, & Heir, 2013).

Currently, the Diagnostic and Statistical Manual (DSM) defines dissociation as “a disruption and/or discontinuity in the normal integration of consciousness, memory, identity, emotion, perception, body representation, motor control, and behavior” (American Psychiatric Association [APA], 2013, p. 291). Clinical presentations of dissociation may include a wide variety of symptoms, including experiences of depersonalization, derealisation, emotional numbing, flashbacks of traumatic events, absorption, amnesia, voice hearing, interruptions in awareness, and identity alteration. Researchers have argued that the use of a single term, “dissociation,” for such phenomenologically distinct experiences can be confusing and that the term dissociation should instead be deconstructed into multiple factors, thus allowing for a more accurate examination of the different phenomenological constructs (Bryant, 2007; Frewen & Lanius, 2015; Spiegel et al., 2013).

Despite the wide range of dissociative phenomenology observed, one underlying theme that spans both current theoretical constructs and observed clinical presentations of dissociation centers around trauma-related altered states of consciousness (TRASC). Putnam (1996) notes succinctly that “The more severe the trauma …, the greater the likelihood that an individual will be driven into an altered state of consciousness” (p. 176). Like the field of dissociation, the study of consciousness has made great theoretical strides, and four dimensions of consciousness, including time, thought, body, and emotion, have been outlined (Thompson & Zahavi, 2007). We have suggested previously that these four dimensions of consciousness are not only relevant but can also be adapted to the study of dissociation, which may help guide our increasing theoretical, neurobiological, and clinical understanding of this phenomenon and provide insight into the phenomenological and neurobiological differences between normal waking consciousness (NWC) and TRASC (Frewen & Lanius, 2015).

Accordingly, this commentary will review the relevance of each of the four dimensions of consciousness to the theoretical, neurobiological, and clinical underpinnings of trauma-related phenomenology in the context of a four-dimensional model (4-D model) outlining a dissociative and a non-dissociative dimension of each of the four dimensions of consciousness (see Frewen & Lanius, 2015). Clinical applications and future research directions relevant to each dimension and for the overall field of trauma-related dissociation will be discussed.

## Summary of the 4-D model

**Fig. 1:**
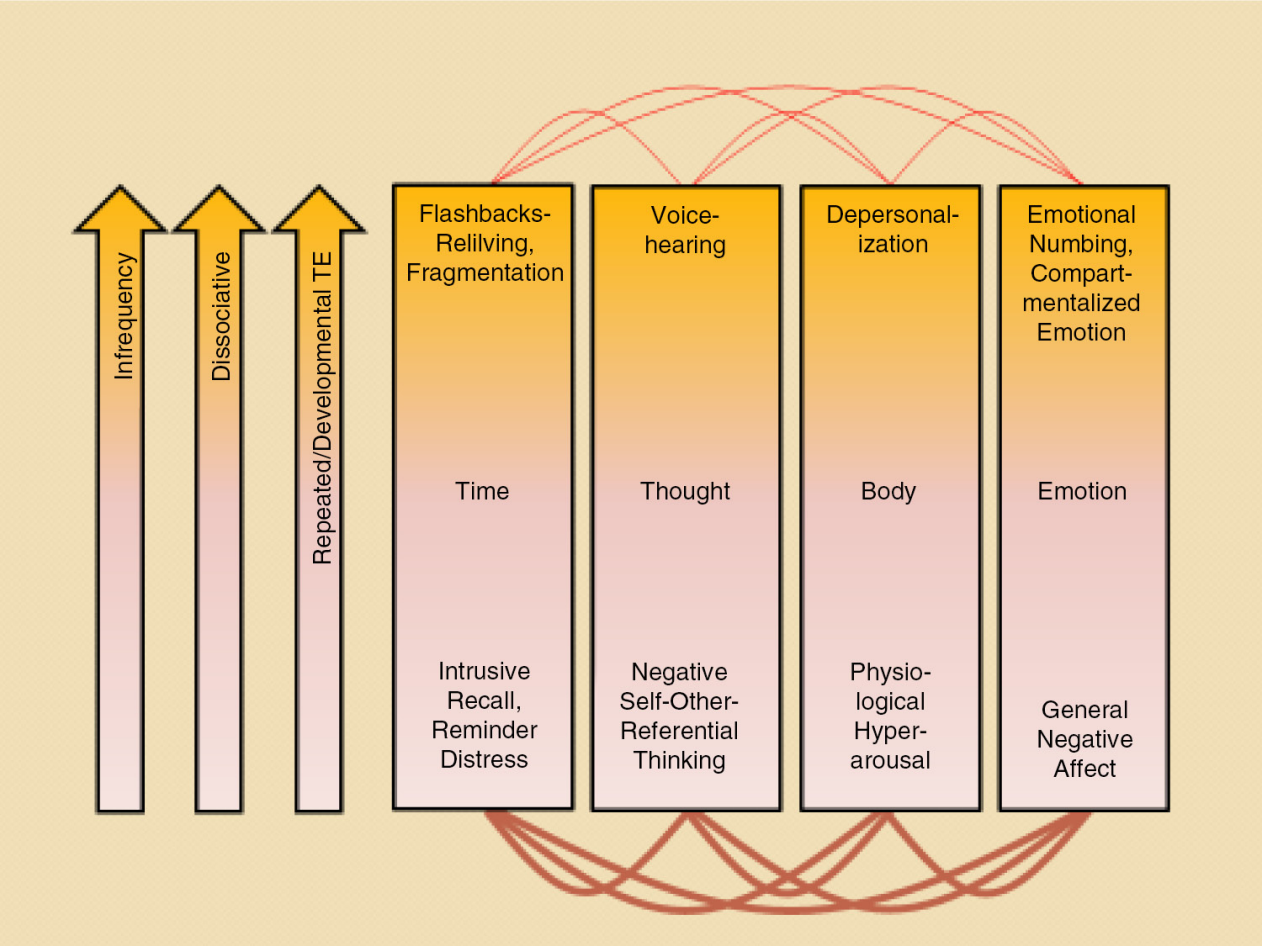
A summary of the 4-D model that categorizes symptoms of trauma-related psychopathology into (1) those that occur within normal waking consciousness and (2) those that are dissociative and are associated with trauma-related altered states of consciousness (TRASC) along four dimensions: (1) time; (2) thought; (3) body; and (4) emotion. The bottom pink part of the boxes indicates non-dissociative processes and normal waking consciousness, whereas the orange part of the boxes denote dissociative processes and TRASC. The first arrow (infrequency) indicates that the experience of TRASC is hypothesized to be less common than presentations of normal waking consciousness given that states of normal waking consciousness, by definition, are the most common phenomenological state of human beings. It should be noted that the four dimensions of consciousness are not mutually exclusive, but may refer to the same phenomena viewed from different perspectives (e.g., depersonalisation can manifest itself both in the dimension body and emotion). Copied with permission from Frewen and Lanius (2015).

The 4D-model classifies symptoms of traumatic stress into (1) those that occur within NWC and (2) those that exhibit a dissociative presentation associated with the experience of TRASC. Experiences of NWC and TRASC can be further classified along four dimensions of consciousness: (1) time (reliving flashbacks [TRASC] versus intrusive memories and reminder distress [NWC]); (2) thought (voice hearing [TRASC] versus negative first-person self-referential thinking [NWC]); (3) body (depersonalization [TRASC] versus hyperarousal [NWC]); and (4) emotion (emotional numbing/shutdown [TRASC] and compartmentalized emotion versus general negative affect [NWC]) (see Fig. 1). It should be noted that the four dimensions of consciousness are not mutually exclusive, but may refer to the same phenomena viewed from different perspectives (e.g., depersonalisation can manifest itself both in the dimension of body and emotion).

The model makes four predictions: (1) the experience of TRASC will be less common than will be presentations of normal waking consciousness given that states of normal waking consciousness, by definition, are the most common phenomenological state of human beings; (2) TRASC are expected to be less intercorrelated than states of normal waking consciousness as the experience of TRASC is frequently experienced as more “compartmentalized”; (3) measures of TRASC are expected to be more strongly correlated with other measures of dissociative symptomatology; and (4) experiences of TRASC are predicted to be more specific to an etiology implicating early life adversity and repeated traumatization. Emerging evidence provides support for these predictions (Frewen et al., 2015; Frewen, Kleindienst, Lanius, & Schmahl, 2014; Frewen & Lanius, 2014). Although a complete exploration of each TRASC is beyond the scope of this article, a brief summary is described below.

## Dimension of time

The flow of consciousness across time is necessary to create an experience of the present, (“now”) in the context of a subjective past and anticipated future. Accordingly, under normal circumstances, time is experienced as continuously moving forward. However, traumatized individuals often relive their traumatic memories through flashbacks and lack the ability to live in the “now,” reflecting a key dissociative process associated with TRASC. Such reliving events are in contrast to intrusive memory recall most frequently associated with reminder distress and not involving an altered state of consciousness or a dissociative process but rather represent a state of normal waking consciousness (Frewen & Lanius, 2015; also see Brewin, 1998).

The associated concepts of episodic memory and autonoesis, the capacity to mentally position oneself in the past or in the future, are particularly relevant to the time dimension of consciousness, as well as the experience of reliving flashbacks. They have been elegantly outlined in the seminal work of Tulving (2005) who notes, “Episodic memory differs from other kinds of memory in that its operations require a self. It is the self that engages in the mental activity that is referred to as mental time travel: there can be no travel without a traveler …” (p. 9). The latter has significant implications for the distinction between reliving versus remembering an experience. Frewen and Lanius (2015) have suggested that while remembering an event, mental time travel is “partial” in that the present self voluntarily directs attention to the past self, thus maintaining awareness of the present self in the present time. In this case, the “I” is proposed to exist in the present self, which outweighs the representation of the past self in past time. In contrast, during a reliving experience, mental time travel occurs “fully,” generally not by choice, and is usually triggered by internal and/or external stimuli that bear some resemblance to a past self-state. In this case, the “I” is thought to inhabit the past self, which is thought to outweigh the presence of the present self, thus lacking a mental time traveler and the ability to voluntarily position oneself in the past or in the future.

Although the neurobiological underpinnings of the time dimension of consciousness are complex, the anterior insula is thought to play an important role in this dimension. Craig (2009) noted that “… the anterior insula supports awareness of the immediate moment with a coherent representation of ‘my feelings’ about ‘that thing’…” (p. 65). The anterior insula has also been shown to be positively correlated with flashback/reliving experiences in PTSD (Hopper, Frewen, Van der Kolk, & Lanius, 2007; Osuch et al., 2001; Whalley et al., 2013), which may reflect collectively altered perception of time, a TRASC or dissociative process, during these experiences.

### Clinical applications

Clinically, it may be critical to strengthen the self among survivors of trauma, in order to facilitate the emergence of a mental time traveler that is able to remember rather than to relive the past. Processes relevant to this development across all four dimensions of consciousness include the encouragement of safe relationships, including the therapeutic relationship, enhancing mindful awareness of the present through mindfulness exercises, emotion regulation, distress tolerance skills, and building capacity for positive affect tolerance. Based on the theoretical assumptions reviewed here, strengthening the sense of self through the use of present-centered therapies (e.g., Ford & Russo, 2006; Frost, Laska, & Wampold, 2014; also see Ford, this issue) in combination with exposure-based treatments (e.g., Cloitre et al., 2010) may be crucial to successfully overcoming severe dissociative flashbacks.

### Future research directions

Future research is required to examine the extent to which an emphasis upon safe relationships, along with the introduction of specific present-centered therapeutic techniques (e.g., Frost et al., 2014; Markowitz et al., 2015) designed to strengthen a present self that is capable of mental time travel, compare and/or add to the benefits relative to standard past/trauma-centered treatments, particularly among individuals who present with significant dissociative flashbacks and TRASC. Here, it will also be critical to develop measures that can adequately assess the sense of self and its role in treatment outcome. Furthermore, an important future research direction includes examining how the capacity for language processing during therapy relates to the quality and frequency of TRASC of time, particularly traumatic flashbacks that are often non-verbal in nature.

With respect to behavioral and biological research, it will be important to examine individuals’ sense of time in relation to the occurrence of dissociative flashbacks and TRASC versus intrusive memories longitudinally through the use of measures that evaluate time perception (e.g., Coelho et al., 2004; Van Wassenhove, Wittmann, Craig, & Paulus, 2011). Monitoring insula activity and patterns of neural connectivity longitudinally in relation to the subjective experience of flashbacks versus intrusive memories will also need to be made a priority in order to further elucidate the neurobiology underlying the consciousness of time in trauma-related psychopathology.

## Dimension of thought

The dimension of consciousness relating to thought relates to the understanding that consciousness is inherently referential, requiring both a subject and an object. In other words, one cannot be conscious unless one is conscious *of something* (Brentano, 1968), which itself necessitates the presence of a conscious being or subject. Consciousness requiring both a subject and an object has been likened to a narrative, which entails perspective (usually first person), a plot, and a structure normally consisting of a beginning, middle, and an end. Psychological trauma may not only affect the perspective of an individual's narrative but also the plot and the structure of the narrative. Although often able to maintain first-person perspective, trauma survivors may exhibit distinctly negative self-referential thinking, including “I am a bad person” or “I do not deserve to live” (e.g., Cox, Resnick, & Kilpatrick, 2014; Foa, Ehlers, Clark, Tolin, & Orsillo, 1999). According to the 4-D model, this type of negative self-referential thinking, during which the first person perspective is maintained, reflects normal waking consciousness (Frewen & Lanius, 2015).

Traumatized individuals may, however, occasionally exhibit alterations in the perspective of their narrative. These alterations can lead survivors to experience voices in the second-person perspective, for example, telling them, “*you* are bad” or “*you* deserve to die,” an experience thought to reflect a dissociative process associated with TRASC (Frewen & Lanius, 2015). When this occurs, the person is no longer the only storyteller of his/her lived experience but rather another or other narrative voice(s) also speak inside his/her head. These voices may present distinctly different goals, motivations, and affects, in the extreme case creating the experience of possessing multiple selves (Frewen & Lanius, 2015). Research in the area of voice hearing has suggested that this phenomenon is elevated significantly in individuals suffering from trauma-related disorders, including in individuals diagnosed with PTSD, dissociative disorders, and borderline personality disorders as compared to patients with other psychiatric disorders (Longden, Madill, & Waterman, 2012); voice hearing is also related to the experience of dissociative symptomatology (e.g., Anketell et al., 2010; Brewin & Patel, 2010; Dorahy et al., 2009) and a history of early life adversity (e.g., McCarthy-Jones, 2011).

Although the neurobiological correlates of voice hearing in trauma-related disorders are yet to be elucidated, a meta-analysis carried out in patients with schizophrenia demonstrated that during voice hearing, brain regions, including Broca's area and Wernicke's area involved in speech production and comprehension, respectively, exhibited brain activation as compared to when patients reported their voices to be absent (Jardri, Pouchet, Pins, & Thomas, 2011). Similar findings were reported in a non-psychiatric group that exhibited voice hearing (Linden et al., 2011). Together, these findings to some degree support the actual “reality” of voice hearing.

### Clinical applications

From a clinical perspective on trauma-related voice hearing, it is crucial to create a shared narrative by identifying the strengths of each voice or self state in the present and by encouraging awareness and communication among different voices or self states, thereby facilitating collaboration between or among distinctly compartmentalized and contradictory goals, motivations, and affects associated with each voice or self state (see Paivio & Pascual-Leone, 2010). The latter is also critical in fostering of self-compassion (also see Kearney et al., 2013), which is sorely lacking in many survivors of chronic trauma due to ongoing conflict among different voices or self states.

### Future research directions

Future research is needed to identify more precisely the neurobiology underlying voice hearing and negative self-referential processing in trauma-related disorders as compared to that underlying voice hearing in psychotic-spectrum and other psychiatric disorders. This will be important to facilitate more accurate diagnosis, thereby guiding the most appropriate treatment interventions. Here, it will also be critical to examine how emotional triggers, for example, positive or negative self-related statements, may affect differently the presentation and underlying neurobiology of voice hearing in trauma-related disorders as compared to psychotic and other psychiatric disorders. Moreover, it will be important to examine the integrity of neural networks, such as the default mode network, which has been associated with an integrated sense of self across time (Lanius, Frewen, Tursich, Jetly, & McKinnon, 2015), pre- and post-treatment interventions that target specifically the creation of a shared narrative between or among voices that may be associated with different self states.

From a clinical perspective, treatment outcome studies that focus specifically on interventions designed to affect voice hearing in trauma-related disorders should also be urgent foci of investigation (see Schwartz, 1995). The use of electroencephalogram (EEG)-based biofeedback may also be an important avenue to target directly the neural circuitry underpinning voice-hearing in trauma-related disorders.

## Dimension of body

The dimension of consciousness relating to the body refers to the belief that thoughts, feelings, and actions originate from the body. As Damasio (1999) described “Whatever happens in your mind happens in time and in space relative to the instant in time your body is in and to the region of space occupied by your body …” (p. 145). However, among individuals who suffer from the aftermath of trauma, the mind/body connection is often severed, leading to the subjective experience of feeling partially or fully detached from one's body, or alternatively, as if one's body does not belong to oneself. These experiences form the core of symptoms of depersonalization, and, accordingly, have long been recognized in many questionnaires tapping dissociation (e.g., Dissociative Experience Scale [Bernstein & Putnam, 1986], Multiscale Dissociation Inventory [Briere, 2002], Multidimensional Inventory of Dissociation [Dell, 2006]) and represent the core of the recently described dissociative subtype of PTSD (Lanius, Brand, Vermetten, Frewen, & Spiegel, 2012; Lanius, Vermetten, et al., 2010; Wolf, Lunney, et al., 2012; Wolf, Miller, et al., 2012; also see Dalenberg & Carlson, 2012). As part of the 4-D model, depersonalization experiences are consistent with TRASC. By contrast, physiological hyperarousal is thought to represent the non-dissociative or normal waking consciousness continuum of the body dimension of consciousness (Frewen & Lanius, 2015).

The neurobiological correlates of depersonalization have been outlined in detail, both in the field of neurology and psychiatry, and are thought to involve activation of the temporoparietal junction (Blanke et al., 2005; Blanke, Ortigue, Landis, & Seeck, 2002; Hopper et al., 2007). In addition, prefrontal inhibition of limbic regions, including the amygdala, has been proposed as underlying the hypo-emotionality and lack of arousal often associated with these states (Lanius, Vermetten, et al., 2010; Sierra & Berrios, 1998). The anterior insula, a brain region that plays an important role in interoceptive awareness, has also been shown to be negatively associated with symptoms of depersonalization and derealisation (Hopper et al., 2007). Recently, it was demonstrated that states of depersonalization can impair the encoding of episodic memory in healthy individuals, a process thought related to altered activation of the posterior hippocampus (Bergouignan, Nyberg, & Ehrsson, 2014). This observation may provide insight into the neural mechanisms underlying dissociative amnesia associated with traumatic experiences that involves depersonalization responses at the time of the trauma and has implications for both the dimension of body and time (also see Dalenberg et al., 2012; Schauer & Elbert, 2010).

### Clinical applications

It is critical for clinicians to understand the subjective experience from which the traumatized individual experiences his/her body and its relation to the surrounding world. Body-scan meditations, intended to facilitate awareness and the monitoring bodily sensations, form a central part of the mindfulness-based stress reduction program developed by Kabat-Zinn (1990) and provide an important means of assessing states of full or partial depersonalization, while at the same time enhancing the capacity for interoceptive awareness and diminishing detachment from bodily states (Follette, Briere, Rozelle, Hopper, & Rome, 2014; Frewen & Lanius, 2015). It is critical to note, however, that body scans must be carried out in a trauma-sensitive way in order to prevent the traumatized individual from becoming overwhelmed during this exercise (see Frewen & Lanius, 2015, for details).

### Future research directions

It will be crucial for future research to examine the effects of body-focused interventions such as body-scan meditations (Kabat-Zinn, 1990) on the neural circuitry underlying interoceptive awareness, including the insula, to investigate whether normalization of the activation of this brain region may mediate the effects of this intervention for depersonalization. For example, detailed investigation of the capacity for interoceptive awareness, where individuals report the rate of their own heart rate, and its relation to anterior insula functioning and emotional detachment will be important, before, during, and after treatments designed to enhance interoceptive awareness (see Critchley, 2004). Moreover, elucidating the relation and underlying neurobiology between states of depersonalization versus hyperarousal and the encoding of episodic memory (see Bergouignan et al., 2014) will need to be a priority in order to gain an increased understanding of the mechanisms underlying amnestic states that frequently follow traumatic encounters (also see Dalenberg et al., 2012) and to elucidate further the relationship between the body and time dimension of consciousness.

## Dimension of emotion

With respect to the dimension of consciousness relating to emotion, theorists have suggested that emotional processing is an essential component in the evolution of consciousness across animal and human species (e.g., Izard, 2007; Panksepp, 1998; Panksepp & Northoff, 2009). Craig (2010) has proposed further that “The unified representation of all salient conditions—encoded as feelings—is in effect a representation of the entirety of the individual … which I refer to as ‘the sentient self’” (p. 569). In the aftermath of trauma, however, it is well documented that emotion dysregulation can range from states of emotional undermodulation during which the individual experiences painful states of fear, anger, guilt, and shame (Miller & Resick, 2007) to states of emotional overmodulation, during which the individual experiences emotional detachment such as states of depersonalization, derealization, emotional numbing, and affective shut-down (Lanius, Frewen, Vermetten, & Yehuda, 2010; Lanius, Vermetten, et al., 2010). Indeed, many traumatized individuals frequently experience intense emotional states to the point that they no longer have the experience of *having* an emotion but rather feel like they *are* the emotion, which according to the 4-D model, would be conceptualized as a state of normal waking consciousness, rather than representing a dissociative process. By contrast, experiences of extreme emotional numbing and affective shutdown and/or significant compartmentalization of emotion to the point where different emotional states are experienced as being felt by a “perceived other” is conceptualized as a dissociative process or TRASC by the 4-D model (Frewen & Lanius, 2015).

The neurobiology underlying the consciousness of emotion is highly complex, and a detailed discussion is beyond the scope of this commentary. However, prefrontal regions, including but not limited to the dorso- and ventromedial prefrontal cortex, perigenual and ventral anterior cingulate and posterior cingulate cortex, inferior parietal lobe, and temporal pole have each been demonstrated to be important to consciousness relating to emotion (Frewen & Lanius, 2015). Notably, emotional numbing symptoms in individuals with PTSD have been associated with decreased amygdala activation while viewing happy faces (Felmingham et al., 2014) and are also negatively correlated with activation in the dorsomedial prefrontal cortex, a brain region involved in self-reflective functioning, during presentation of standardized positive and negative scripts (Frewen, Dozois, Neufeld, et al., 2012). Taken together, these findings suggest that the neural circuitry underlying the consciousness of emotion is altered as a function of emotional numbing in PTSD. Research examining the neural correlates of compartmentalization of emotion, however, is yet to be conducted. Nonetheless, an emerging body of literature points towards differential brain activation patterns in response to traumatic memories and emotional face processing in patients with dissociative identity disorder (DID), depending upon the dissociated self-state that is present at the time of assessment (e.g., Reinders et al., 2006; Reinders et al., 2014; Schlumpf et al., 2013).

### Clinical applications

Clinical efforts to assist individuals in overcoming emotional numbing and affective shutdown may center around assisting the traumatized individual to shift out of his/her shut-down state in order to be able to feel a full range of emotions, particularly pleasure and joy (also see Etter, Gauthier, McDade-Montez, Cloitre, & Carlson, 2013; Frewen, Dean, & Lanius, 2012; Frewen, Dozois, & Lanius, 2012). Since subjective conscious emotional experience appears to be based on the perception of physical sensations and bodily states resulting from endocrine, autonomic nervous system, musculoskeletal, and vestibular influences (Barrett, Mesquita, Ochsner, & Gross, 2007; Damasio, 1996; James, 1884), mapping the physical bodily sensations related to specific emotions through the use of body scan meditations (Kabat-Zinn, 1990) can be helpful in helping individuals identify what feelings and emotions they experience, thus enabling them to overcome symptoms of emotional numbing and shutdown. Further support for the latter approach stems from a recent cross-cultural study that demonstrated specific maps of bodily sensations in association with the experience of different emotions (Nummenmaa, Glerean, Hari, & Hietanen, 2014). In the case of severely compartmentalized emotional states, treatment designed to facilitate collaboration and awareness between or among distinctly different dissociated parts of the self (exhibiting distinct emotional states), as described above under voice hearing, is also an important goal.

### Future research directions

It is imperative that future research efforts in the area of consciousness of emotion compare directly the capacity of (1) traumatized individuals with frequent symptoms of emotional numbing and shutdown and (2) traumatized individuals that solely exhibit general negative affect to identify specific maps of bodily sensations and to explore the relation of these maps to the experience of different emotional experience. These findings will then need to be related not only to the underlying neural deficits of emotion processing but also to treatment outcomes. Moreover, investigating how specific neural networks involved in the consciousness of emotion can be directly targeted through the use of real-time fMRI or EEG-based biofeedback in conjunction with pharmacotherapeutic and psychotherapeutic approaches will be an important avenue for future investigation.

## Concluding remarks

In summary, this commentary describes a 4-D model that categorizes symptoms of trauma-related psychopathology into (1) those that occur within normal waking consciousness and (2) those that are dissociative and associated with TRASC along four dimensions of consciousness: (1) time; (2) thought; (3) body; and (4) emotion. Conceptualizing TRASC across these four dimensions has transdiagnostic implications for trauma-spectrum disorders involving dissociation and is relevant to efforts to identify key factors related to the development and maintenance of trauma-related symptoms described both in the DSM and the International Classification of Disease. It will be crucial for future research to examine directly these symptoms along each dimension of consciousness and to identify further their neurobiological and physiological underpinnings, and to determine their role in treatment outcome (also see Sar, 2014). This work, guided by the 4-D model as well as numerous existing models of dissociation, has the potential to advance collectively the important and often neglected field of dissociation.

## Supplementary Material

Trauma-related dissociation and altered states of consciousness: a call for clinical, treatment, and neuroscience research

Trauma-related dissociation and altered states of consciousness: a call for clinical, treatment, and neuroscience research

Trauma-related dissociation and altered states of consciousness: a call for clinical, treatment, and neuroscience research

Trauma-related dissociation and altered states of consciousness: a call for clinical, treatment, and neuroscience research

Trauma-related dissociation and altered states of consciousness: a call for clinical, treatment, and neuroscience research

Trauma-related dissociation and altered states of consciousness: a call for clinical, treatment, and neuroscience research
