# Supplementary material for: Trauma-related dissociation and altered states of consciousness: a call for clinical, treatment, and neuroscience research
Source: Eur J Psychotraumatol. 2015 May 19;6:10.3402/ejpt.v6.27905. doi: 10.3402/ejpt.v6.27905 (PMC4439425; doi:10.3402/ejpt.v6.27905)
Supplement: Trauma-related dissociation and altered states of consciousness: a call for clinical, treatment, and neuroscience research [file EJPT-6-27905-s001.pdf]

Dissociation et états altérés de la conscience liés à un traumatisme: Appel pour la recherche clinique, traitements et neurosciences

Ruth A. Lanius

L'objectif principal de ce commentaire est de décrire la dissociation liée à un traumatisme et les états altérés de la conscience dans le contexte d'un modèle à 4 dimensions récemment proposé (Frewen & Lanius, 2015). Ce modèle classe les symptômes de psychopathologie liés à un traumatisme en i) ceux qui se produisent dans la conscience de veille normale; et ii) ceux qui sont dissociatifs et sont associés à des états modifiés de conscience liés au traumatisme autour de quatre dimensions: a) le temps; b) la pensée; c) le corps; et d) les émotions. Les applications cliniques et orientations futures de la recherche pertinentes pour chaque dimension sont également discutées. Conceptualiser les états modifiés de la conscience liés au traumatisme à travers les dimensions du temps, de la pensée, du corps et des émotions a des implications transdiagnostic pour les troubles liés à un traumatisme comme décrits à la fois dans le manuel diagnostique et statistique (DSM) et les classifications internationales des maladies (ICD). Le modèle à 4 dimensions fournit un cadre, guidé par les modèles existants de dissociation, pour les futures recherches voulant examiner les fondements phénoménologiques, neurobiologiques, et physiologiques des dissociations liées à un traumatisme.

Mots-clés: dissociation ; conscience ; sensibilisation intéroceptive ; sous-type dissociatif ; émotion ; cortex cingulaire antérieur, insula, ESPT complexe

Citation: European Journal of Psychotraumatology 2015, **6**: 27905 - http://dx.doi.org/10.3402/ejpt.v6.27905
