# Supplementary material for: Trauma-related dissociation and altered states of consciousness: a call for clinical, treatment, and neuroscience research
Source: Eur J Psychotraumatol. 2015 May 19;6:10.3402/ejpt.v6.27905. doi: 10.3402/ejpt.v6.27905 (PMC4439425; doi:10.3402/ejpt.v6.27905)
Supplement: Trauma-related dissociation and altered states of consciousness: a call for clinical, treatment, and neuroscience research [file EJPT-6-27905-s002.pdf]

Trauma-bezogene Dissoziationen und veränderte Bewußtseinszustände: ein Aufruf für Klinische-, Behandlungs- und Neurowissenschaftliche Forschung

Ruth A. Lanius

Ziel dieses Kommentars ist es trauma-bezogene Dissoziationen und veränderten Bewußtsseinszustände im Kontext eines vier-dimensionalen Modells (Frewen & Lanius, 2015), welches unlängst vorgeschlagen wurde, zu beschreiben. Dieses Modell kategorisiert Symptome traumabezogener Psychopathologie in i) jene, die im üblichen Wachheitszustand auftreten, ii) jenen, die dissozierend sind und mit trauma-verbundenen veränderten Bewußtseinsständen verbunden sind: a) Zeit, b) Gedanken, c) Körper, und d) Gefühle. Klinische Anwendungen und zukünftige Forschungsrichtungen in Bezug auf jede Dimension werden diskutiert. Das Vier-Dimensionen Modell stellt einen Rahmen zur Verfügung, geleitet von existierenden Modellen für die Dissoziation. Für zukünftige Forschung überprüft es phänomenologische, neurobiologischen und physiologische Grundlagen der trauma-bezogenen Dissoziationen.

Schlüsselwörter: Dissoziation, Bewußtsein, interozeptives Bewußtsein, dissoziative Subtypen, Gefühl, anteriores Cingulum, Insula, Komplexe PTBS

Citation: European Journal of Psychotraumatology 2015, **6**: 27905 - http://dx.doi.org/10.3402/ejpt.v6.27905
