# Supplementary material for: Trauma-related dissociation and altered states of consciousness: a call for clinical, treatment, and neuroscience research
Source: Eur J Psychotraumatol. 2015 May 19;6:10.3402/ejpt.v6.27905. doi: 10.3402/ejpt.v6.27905 (PMC4439425; doi:10.3402/ejpt.v6.27905)
Supplement: Trauma-related dissociation and altered states of consciousness: a call for clinical, treatment, and neuroscience research [file EJPT-6-27905-s003.pdf]

Traumás disszociáció és módosult tudatállapotok: Klinikai, terápiás és idegtudományi kutatások

Ruth A. Lanius

A tanulmány elsődleges célja a traumás disszociáció és traumával összefüggő módosult tudatállapotok leírása az újonnan megfogalmazott 4 dimenziós modell mentén (Frewen & Lanius, 2015). Ez a modell a tüneteket az alapján kategorizálja, hogy azok i) normál éber tudatállapotban vagy ii) disszociatív, illetve módosult tudatállapotban jelentkeznek. A tünetek az alábbi dimenziók mentén helyezhetők el: a) idő, b) gondolkodás, c) test és d) érzelmek. A traumával kapcsolatos módosult tudatállapotok konceptualizálása az idő, gondolkodás, test és érzelmek mentén transzdiagnosztikus implikációkkal bír, mind a DSM, mind a BNO számára. A 4 dimenziós modell jó elméleti keretül szolgál – a disszociáció meglévő modelljeivel együtt – a traumás disszociáció neurobiológiai és fiziológiai kutatásaihoz.

Kulcsszavak: disszociáció; tudat; interoceptív tudatosság; disszociatív altípus; érzelem; anterior cinguláris kéreg, inzula; komplex PTSD

Citation: European Journal of Psychotraumatology 2015, **6**: 27905 - http://dx.doi.org/10.3402/ejpt.v6.27905
