# Supplementary material for: Trauma-related dissociation and altered states of consciousness: a call for clinical, treatment, and neuroscience research
Source: Eur J Psychotraumatol. 2015 May 19;6:10.3402/ejpt.v6.27905. doi: 10.3402/ejpt.v6.27905 (PMC4439425; doi:10.3402/ejpt.v6.27905)
Supplement: Trauma-related dissociation and altered states of consciousness: a call for clinical, treatment, and neuroscience research [file EJPT-6-27905-s004.pdf]

Disociación relacionada con el trauma y estados alterados de conciencia: una llamada a la investigación clínica, de tratamiento y de neurociencia

Ruth A. Lanius

El objetivo principal de este artículo es describir la disociación relacionada con el trauma y los estados alterados de conciencia en el contexto de un modelo de cuatro dimensiones que se ha propuesto (Frewen & Lanius, 2015). Este modelo categoriza los síntomas de la psicopatología relacionada con el trauma en i) los que ocurren dentro de un estado consciente de vigilia normal; y ii) los que son disociativos y están asociados con estados de conciencia alterados relacionados con el trauma en cuatro dimensiones: a) tiempo; b) pensamiento; c) cuerpo; y d) emoción. También se habla de las aplicaciones clínicas y las futuras líneas de investigación que son relevantes en cada dimensión. Conceptualizar los estados alterados de conciencia relacionados con el trauma a través de las dimensiones de tiempo, pensamiento, cuerpo y emoción tiene implicaciones transdiagnósticas para los trastornos relacionados con el trauma descritos tanto en el manual diagnóstico estadístico como en la clasificación internacional de enfermedades. El modelo de cuatro de dimensiones aporta una estructura determinada por los modelos existentes de disociación a las futuros estudios que examinen las claves fenomenológicas, neurobiológicas y psicológicas de la disociación relacionada con el trauma.

Palabras clave: disociación; conciencia; conciencia interoceptiva; subtipo disociativo; emoción; cingulado anterior, ínsula; TEPT complejo

Citation: European Journal of Psychotraumatology 2015, **6**: 27905 - http://dx.doi.org/10.3402/ejpt.v6.27905
