# Supplementary material for: Trauma-related dissociation and altered states of consciousness: a call for clinical, treatment, and neuroscience research
Source: Eur J Psychotraumatol. 2015 May 19;6:10.3402/ejpt.v6.27905. doi: 10.3402/ejpt.v6.27905 (PMC4439425; doi:10.3402/ejpt.v6.27905)
Supplement: Trauma-related dissociation and altered states of consciousness: a call for clinical, treatment, and neuroscience research [file EJPT-6-27905-s005.pdf]

Travma alakalı Dissosiyasyon ve Farklı Bilinç Halleri: Klinik, Tedavi, ve Sinirbilim Araştırması için Bir Çağrı

Ruth A. Lanius

Bu açıklamanın amacı, travma alakalı dissosiyasyonu ve farklı bilinç halleri yakın zamanda önerilen 4-Boyutlu Model bağlamı içerisinde tanımlamaktır (Frewen& Lanius, 2015). Bu model travma ile alakalı psikopatoloji semptomlarını i) normal uyanık bilinçte oluşan; ve ii) dissosiyatif olan ve dört boyutla birlikte travma alakalı bilinç değişimleri ile ilgili olan: a) zaman; b) düşünce; c)beden; ve d) duygu şeklinde kategorize eder. Klinik uygulamalar ve her boyutla ilgili gelecek araştırma yönergeleri de tartışılmaktadır. Travma-alakalı bilinç durumlarını zaman, düşünce, beden, ve duygu süresince kavramsallaştırma hem Ruhsal Bozuklukların Tanısal ve İstatistiksel El Kitabı'nda hem de Hastalıkların Uluslararası Sınıflandırılmasında tanımlanan travma alakalı rahatsızlıklar için olası transdiagnostik sonuçlara sahiptir. 4 Boyutlu Model, travma alakalı dissosiyasyonun olgusal, nörobiyolojik ve fizyolojik destekleyicilerini araştırmaya yönelik gelecek çalışmalar için, var olan dissosiyasyon modelleri tarafından yönlendirilen bir çerçeve sağlamaktadır.

Anahtar Kelimeler: Dissosiyasyon; bilinç; duyarlı farkındalık; dissosiyatif alt grup; duygu; singulat anterior, insula; karmaşık TSSB

Citation: European Journal of Psychotraumatology 2015, **6**: 27905 - http://dx.doi.org/10.3402/ejpt.v6.27905
