# Supplementary material for: Trauma-related dissociation and altered states of consciousness: a call for clinical, treatment, and neuroscience research
Source: Eur J Psychotraumatol. 2015 May 19;6:10.3402/ejpt.v6.27905. doi: 10.3402/ejpt.v6.27905 (PMC4439425; doi:10.3402/ejpt.v6.27905)
Supplement: Trauma-related dissociation and altered states of consciousness: a call for clinical, treatment, and neuroscience research [file EJPT-6-27905-s006.pdf]

Disocijacija povezana sa traumom i izmenjena stanja svesti: poziv za klinička, terapijska i istraživanja u oblasti neuronauka

Ruth A. Lanius

Primarni cilj ovog komentara je da opiše disocijaciju povezanu sa traumom i izmenjena stanja svesti u kontekstu 4-orodimenzionalnog modela koji je nedavno predstavljen (Frewen & Lanius, 2015). Ovaj model kategorizuje simptome psihopatologije povezane sa traumom na sledeći način: i) one koji se dešavaju pri normalnoj budnosti i ii) disocijativne ili one povezane sa izmenjenim stanjima svesti povezanim sa traumom u 4 dimenzije: a) vreme, b) misli, c) telo i d) emocije. Diskutuju se i klinička primenljivost i budući istraživački pravci. Konceptualizovanje izmenjinih stanja svesti u vezi sa traumom kroz 4 dimenzije vreme, mišljenje, telo i emocije ima transdijagnostičku primenu za poremećaje povezane sa traumom i u dijagnostičkom i statističkom priručniku i u Međunarodnoj klasifikaciji bolesti. 4-orodimenzionalni model obezbeđuje okvir, vođen postojećim modelom disocijacije, za buduća istraživanja koja će ispitivati fenomenološke, neurobiološke i fiziološke osnove disocijalcije povezane sa traumom.

Ključne reči: disocijacija; svesnost; interoceptivna budnost; disocijativni podtip; emocije; prednji cingulatni; insula; kompleksni PTSP

Citation: European Journal of Psychotraumatology 2015, **6**: 27905 - **http://dx.doi.org/10.3402/ejpt.v6.27905**
